# Supplementary material for: Association between oral microbiome and seven types of cancers in East Asian population: a two-sample Mendelian randomization analysis
Source: Front Mol Biosci. 2023 Nov 21;10:1327893. doi: 10.3389/fmolb.2023.1327893 (PMC10702768; doi:10.3389/fmolb.2023.1327893)
Supplement: Supplementary file 1 [file DataSheet1.ZIP › Supplementary Materials/MR plots for tongue/tongue═╝/Pancreatic cancer/╥╚╧┘░⌐_╔╕╤í╜ß╣√/pheno.2888_to_pancreatic cancer_leave_one_out.pdf]

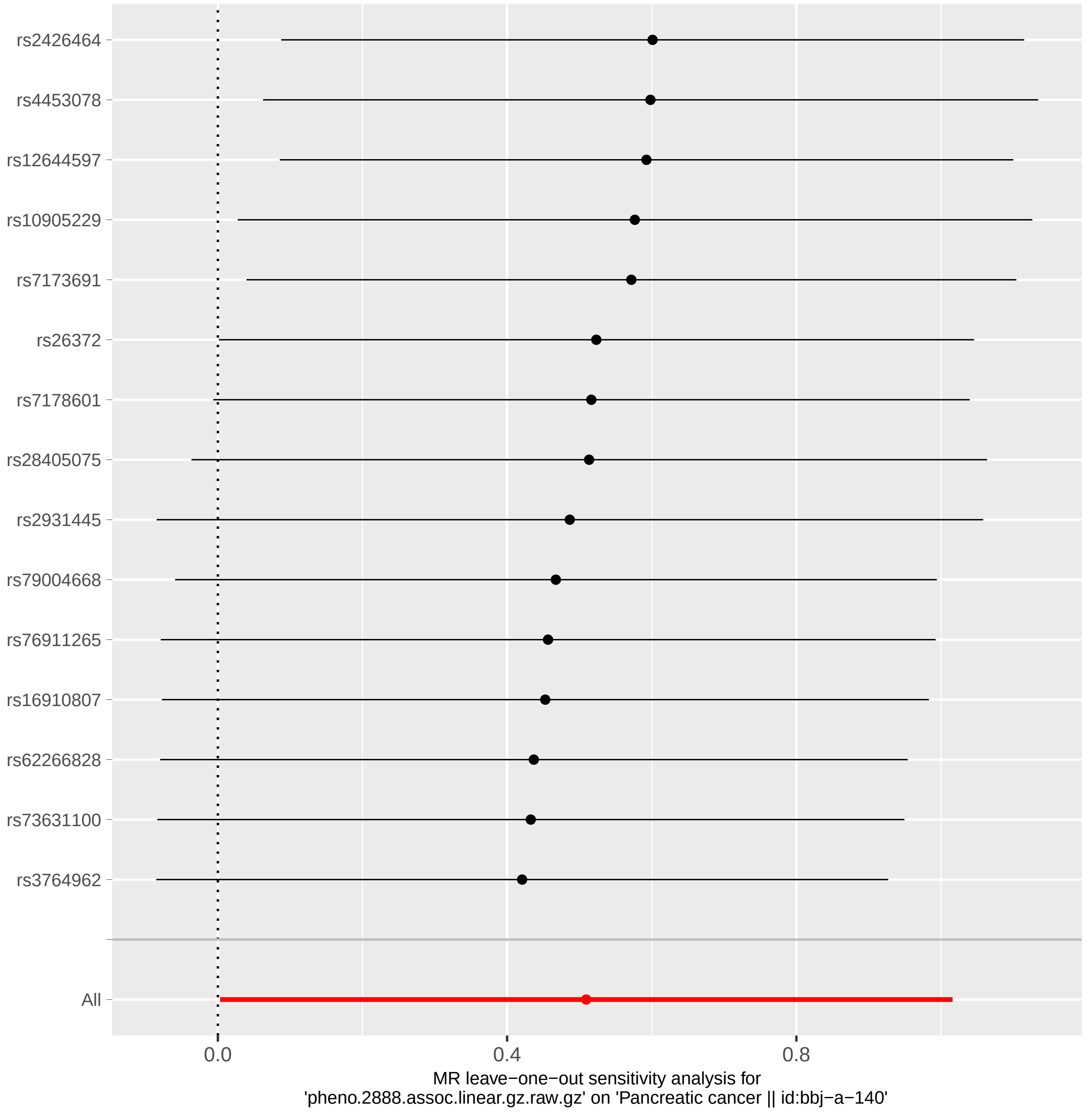

rs2426464
rs4453078
rs12644597
rs10905229
rs7173691
rs26372
rs7178601
rs28405075
rs2931445
rs79004668
rs76911265
rs16910807
rs62266828
rs73631100
rs3764962
All
0.0
0.4
0.8
MR leave−one−out sensitivity analysis for
'pheno.2888.assoc.linear.gz.raw.gz' on 'Pancreatic cancer || id:bbj−a−140'
